# Supplementary material for: Transforming biorefinery designs with ‘Plug-In Processes of Lignin’ to enable economic waste valorization
Source: Nat Commun. 2021 Jun 23;12:3912. doi: 10.1038/s41467-021-23920-4 (PMC8222318; doi:10.1038/s41467-021-23920-4)
Supplement: Supplementary file 1 — Supplementary Information [file 41467_2021_23920_MOESM1_ESM.pdf]

Supplementary information

Transforming Biorefinery Designs with 'Plug-In Processes of Lignin' to Enable Economic Waste Valorization

Zhi-Hua Liu [1, 2], Naijia Hao [3], Yun-Yan Wang [3], Chang Dou [4], Furong Lin [1, 2], Rongchun Shen [5], Renata Bura [4], David B. Hodge [6], Bruce E Dale [7], Arthur J. Ragauskas [3, 8, 9], Bin Yang [5], Joshua S. Yuan [1, 2 *]

[1] Synthetic and Systems Biology Innovation Hub (SSBiH), Texas A&M University, College Station, TX 77843, USA

[2] Department of Plant Pathology and Microbiology, Texas A&M University, College Station, TX 77843, USA

[3] Department of Chemical & Biomolecular Engineering, University of Tennessee, Knoxville, TN 37996, USA

[4] School of Environmental and Forest Sciences, University of Washington, Seattle, WA 98195, USA

[5] Bioproducts, Sciences, and Engineering Laboratory, Department of Biological Systems Engineering, Washington State University, Richland, WA 99354, United States

[6] Chemical and Biological Engineering Department, Montana State University, Bozeman, MT 59717, USA

[7] Biomass Conversion Research Laboratory, Department of Chemical Engineering and Materials Science, Michigan State University, East Lansing, MI 48910, USA.

[8] Biosciences Division, Oak Ridge National Laboratory, Oak Ridge, TN 37830, USA

[9] Department of Forestry, Wildlife and Fisheries, Center for Renewable Carbon, The University of Tennessee Institute of Agriculture, TN 37996-2200

*Corresponding contributor. E-mail: *syuan@tamu.edu*

Supplementary Methods

Bacterial strain and medium preparation

Engineered *Pseudomonas putida* KT2440 strain was stored on the Luria-Bertani plate containing 1.5% agar. For seed culture, a single colony on Luria-Bertani plate was selected and inoculated into 20 ml Luria-Bertani broth, and then the seed culture was grown at 28 °C and 200 rpm for about 18 h. Cell growth was monitored by measuring the optical densities at 600 nm. 1 ml culture solution was then transferred into 100 ml M9 mineral medium supplemented with 20 g/l glucose and 1.0 g/l NH_4Cl, and cultivated at 28 °C, 200 rpm for 24 h. [1]

100 ml seed medium contains 20 g/l glucose, 1.0 g/l NH_4Cl, 10 ml 10X Basal salts, and 1 ml 100X Mg/Ca/B1/Goodies mixture. For the preparation of Basal salts, 30 g KH_2PO_4, 60 g $NaHPO_4$, and 5 g NaCl were mixed with ddH_2O to make into 1 l solution. Stock salt solution was composed of 22.94 g/l $MgCl_2 \cdot 6H_2O$, 2.0 g/l $CaCO_3$, 4.5 g/l $FeSO_4 \cdot 7H_2O$, 1.44 g/l $ZnSO_4 \cdot 7H_2O$, 0.85 g/l $MnSO_4 \cdot H_2O$, 0.25 g/l $CuSO_4 \cdot 5H_2O$, 0.24 g/l $CoCl_2 \cdot 6H_2O$, 0.06 g/l H_3BO_3, and 51.3 ml HCl. For 100X Mg/Ca/B1/Goodies mixture, 500 ml stock salt solution, 3.009 g $MgSO_4$, and 25 ml 1% $FeSO_4$ was mixed to make 1.0 l concentrated Goodies. 250 ml concentrated Goodies were then mixed well with 200 ml 1 M $MgSO_4$, 10 ml 1 M $CaCl_2$, and 10 ml 10 mM thiamine to make 1.0 l 100X Mg/Ca/B1/Goodies mixture.

Nuclear magnetic resonance analysis

The two-dimensional (2D) heteronuclear single quantum coherence (HSQC) nuclear magnetic resonance (NMR) spectra of all lignin samples were obtained with a Varian 500 MHz NMR spectrometer with the “gradient HSQCAD” mode. 30~50 mg lignin sample was dissolved in 0.6 ml dimethylsulfoxide (DMSO)-d_6. The gradient-enhanced HSQC with adiabatic pulses (gHSQCAD) mode was employed using the following parameters: 1.0 pulse delay, 32 scans, 1024 data points for ^{1}H, and 256 increments for ^{13}C. The central solvent peak (δC/δH=39.5/2.49 ppm) was used for reference. [2-4]

^{31}P NMR analysis of all lignin samples was performed on a Varian 500 MHz

spectrometer. 20-25 mg lignin sample was dissolved in 0.7 ml stock solution of pyridine/$CDCl_3$ (v/v=1.6/1) containing 1.25 mg/ml $Cr(acac)_3$ and 2.5 mg/ml internal standard *endo*-N-hydroxy-5-norbene-2,3-dicarboxylic acid imide (NHND). The vial was shaken to dissolve lignin completely. Prior to the NMR analysis, 70 μl phosphorylating reagent 2-chloro-4,4,5,5-tetramethyl-1,3,2-dioxaphospholane was added to the vial and mixed well. Quantitative ^{31}P NMR spectra of all lignin samples were carried out using an inverse-gated decoupling pulse sequence, 90° pulse angle, 1.2 s acquisition time, 25 s pulse delay, and 64 scans. [2-4]

Gel-permeation chromatography (GPC) analysis

Lignin sample was acetylated with acetic anhydride/pyridine (1/1, v/v) for 24 h in a sealed flask under an inert atmosphere. After 24 h, the solution was diluted with 20 ml of ethanol and stirred for 30 min. The solvents were then removed with a rotary evaporator followed by drying in a vacuum oven at 40°C. Prior to GPC analysis, the acetylated lignins were dissolved in tetrahydrofuran (1.0 mg/ml) and filtered through a 0.45 μm filter. The molecular weight analysis of lignin samples was conducted by using an Agilent GPC SECurity 1200 system equipped with three Waters Styragel columns (HR1, HR2, and HR6), an Agilent refractive index detector, and an Agilent UV detector (270 nm). Tetrahydrofuran was used as the mobile phase with a flow rate of 1.0 ml/min. A standard polystyrene sample was used for calibration. [5, 6]

Techno-economic analysis of biological lignin valorization

Techno-economic analysis of biological lignin valorization was carried out to evaluate the 'plug-in processes of lignin (PIPOL)' with the integration of leading pretreatment technologies. The production process of polyhydroxyalkanoates (PHAs) includes lignin treatment, PHA fermentation, cell recovery, and PHA extraction sections (Supplementary Fig. 12).

The conditioning process is used to modify the waste stream, which contains lignin to be appropriate for PHA fermentation (Supplementary Fig. 13). The soluble lignin stream from the solubilization has a higher pH value, which is not suitable for bacteria

growth. Thus, a neutralization step is needed to improve the efficiency of substrate utilization within the conditioning system. Sulfuric acid is fed into the system in order to adjust the pH value of soluble lignin stream.

In the PHA fermentation process (Supplementary Fig. 14), the lignin and residual sugar in soluble lignin feed stream is converted to PHAs using engineered *P. putida* KT2440. *P. putida* KT2440 is a natural aromatic-catabolizing organism, which demonstrates their aromatic metabolic pathways converting both aromatic compounds and lignin-enriched streams into PHAs. In order to provide a required 10% inoculum volume back to the production fermentors, 10% of the conditioned slurry is split off to seed production. The seed culture is operated in a batch mode with a 12-hour batch time and an additional 12-hour turnaround time.

The cell harvesting operation is concerned mainly in collecting *P. putida* KT2440 cell biomass from the fermentation broth by using filter press and vacuum dryer (Supplementary Fig. 15). The dried and powdered *P. putida* KT2440 cells are produced and ready for PHA extraction. Cell harvesting involves cell precipitation from the broths, filter pressing and vacuum drying for dewatering and grinding as final step prior to PHA extraction

The extraction operation of PHAs is responsible for the recovery of the PHAs produced intracellularly in *P. putida* KT2440 (Supplementary Fig. 16). Cell powders from fermentation are being processed. PHA extraction is commenced with the disruption of the cell powders by introducing ethyl acetate in a continuously stirred batch tank. Two cell precipitation tanks are needed to accommodate disruption of the cell powders. After cell disruption, aqueous solution is introduced into the filter press and centrifuge for subsequent removal of insoluble solids. Ethyl acetate containing PHA flocculates is then subjected for precipitation. After precipitation, PHA polymers are separated using a 2 μm filter press. With the required solvent in the extraction process, a solvent recovery is definitely a must. After PHA polymers are dewatered, it will be washed to remove residual salts. After that, PHAs will be dried in a vacuum dryer. Dried PHA is ready for storage and/or marketing.

Calculation of sugar conversion and yield

Mass balance was carried out in the whole fractionation process including pretreatment, solubilization and enzymatic hydrolysis of corn stover biomass. Sugar conversion in the enzymatic hydrolysis was calculated as follows:

$$Glucan\ conversion\ (\%) = \frac{[Glucose_{hydrolysate}]}{[Glucan_{solid\ used\ in\ hydrolysis} \times \frac{180}{162}]} \times 100\% \qquad (1)$$

$$Xylan\ conversion\ (\%) = \frac{[Xylose_{hydrolysate}]}{[Xylan_{solid\ used\ in\ hydrolysis} \times \frac{150}{132}]} \times 100\% \qquad (2)$$

Sugar yield in the whole fractionation process was calculated as follows:

$$Glucose\ yield\ (\%) = \frac{[Glucose_{liquid\ of\ pretreatment} + Glucose_{liquid\ of\ solubilization} + Glucose_{hydrolysate}]}{[Glucan_{feedstock} \times \frac{180}{162}]} \times 100\% \qquad (3)$$

$$Xylose\ yield\ (\%) = \frac{[Xylose_{liquid\ of\ pretreatment} + Xylsoe_{liquid\ of\ solubilization} + Xulose_{hydrolysate}]}{[Xylan_{feedstock} \times \frac{150}{132}]} \times 100\% \qquad (4)$$

180/132 represents the stoichiometric factor of converting glucose to glucan, while 150/132 represents the stoichiometric factor of converting xylose to xylan.

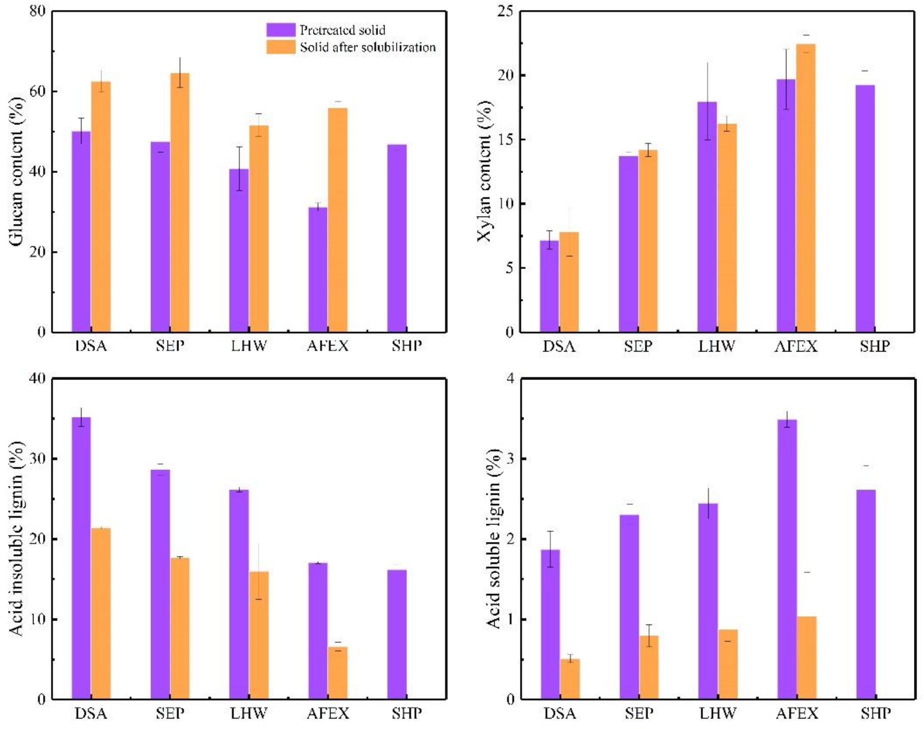

Supplementary Fig. 1 Glucan, xylan and lignin transformation in corn stover after leading pretreatment and solubilization. DSA, dilute sulfuric acid pretreatment; SEP, steam explosion pretreatment; LHW, liquid hot water pretreatment; AFEX, ammonia fiber expansion; SHP, sodium hydroxide pretreatment. Error bars represent the standard deviation.

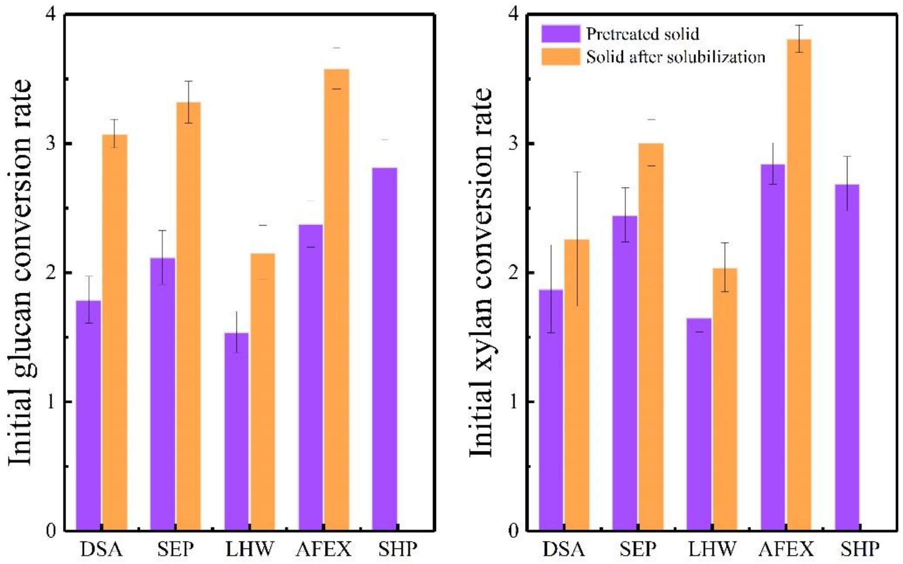

Supplementary Fig. 2 Initial glucan and xylan conversion rate in the enzymatic hydrolysis. 1% solid loading was employed in this enzymatic hydrolysis. The solids used in enzymatic hydrolysis were obtained from leading pretreatment and the solubilization. Initial glucan/xylan conversion rate (% per hour) is calculated based on the first 12 h hydrolysis. DSA, dilute sulfuric acid pretreatment; SEP, steam explosion pretreatment; LHW, liquid hot water pretreatment; AFEX, ammonia fiber expansion; SHP, sodium hydroxide pretreatment. Error bars represent the standard deviation.

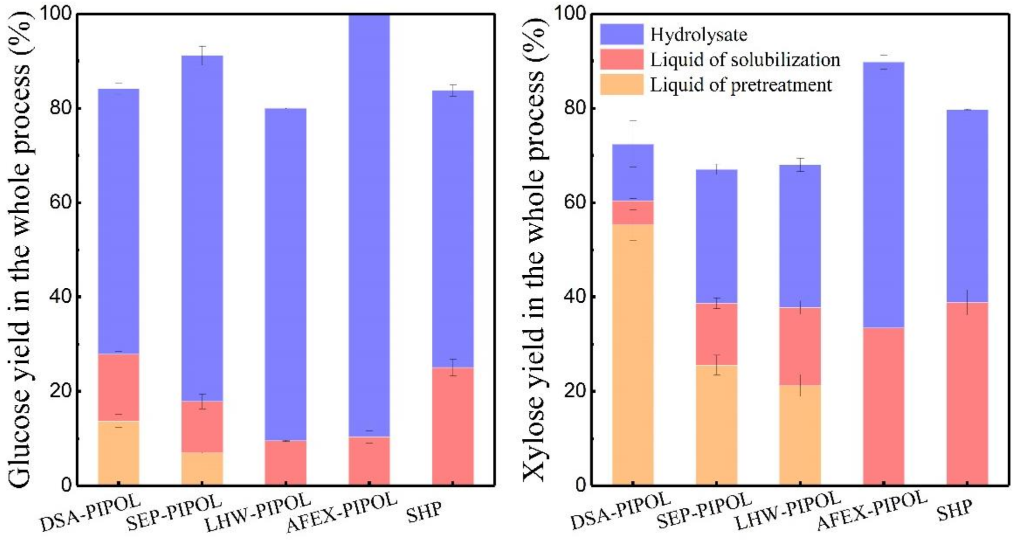

Supplementary Fig. 3 The sugar yields in the whole process of leading pretreatment, solubilization and enzymatic hydrolysis. PIPOL, ‘plug-in processes of lignin’; DSA, dilute sulfuric acid pretreatment; SEP, steam explosion pretreatment; LHW, liquid hot water pretreatment; AFEX, ammonia fiber expansion; SHP, sodium hydroxide pretreatment. Hydrolysate represents the liquid stream of enzymatic hydrolysis. The conditions of leading pretreatment were provided in Supplementary Table 1. Error bars represent the standard deviation.

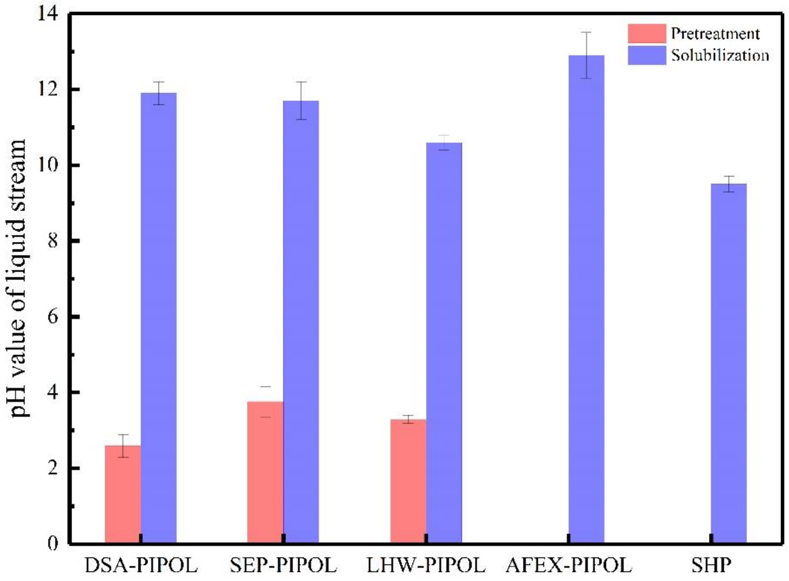

Supplementary Fig. 4 The pH value of the liquid stream from leading pretreatment and solubilization. DSA, dilute sulfuric acid pretreatment; SEP, steam explosion pretreatment; LHW, liquid hot water pretreatment; AFEX, ammonia fiber expansion; SHP, sodium hydroxide pretreatment; PIPOL, Plug-in processes of lignin. Error bars represent the standard deviation.

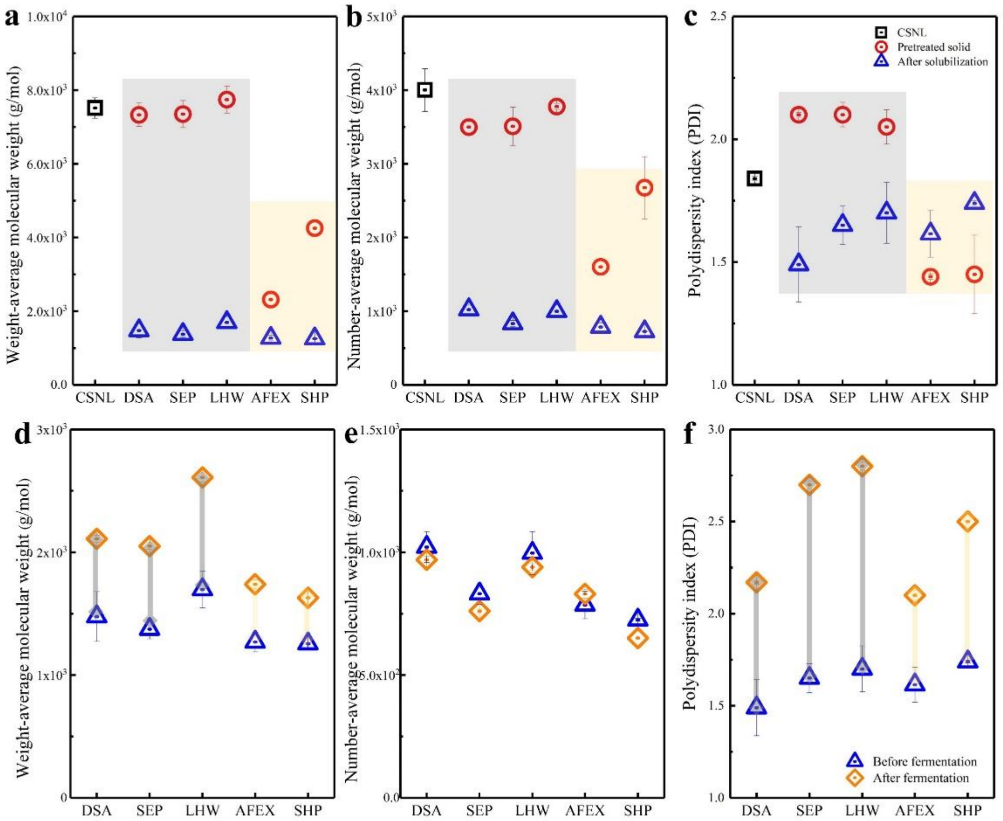

Supplementary Fig. 5 The molecular weight of the lignin samples. a, b and c represents weight-average molecular weight, number-average molecular weight and polydispersity index of the lignin samples, respectively. These lignin samples were from corn stover native lignin (CSNL), the pretreated solid of leading pretreatment, and the soluble lignin after the solubilization. d, e and f represents weight-average molecular weight, number-average molecular weight and polydispersity index of the lignin samples obtained before and after fermentation, respectively. DSA, dilute sulfuric acid pretreatment; SEP, steam explosion pretreatment; LHW, liquid hot water pretreatment; AFEX, ammonia fiber expansion; SHP, sodium hydroxide pretreatment. Gray mark represents the acidic pretreatment technologies, while yellow mark represents the alkaline pretreatment technologies. Error bars represent the standard deviation.

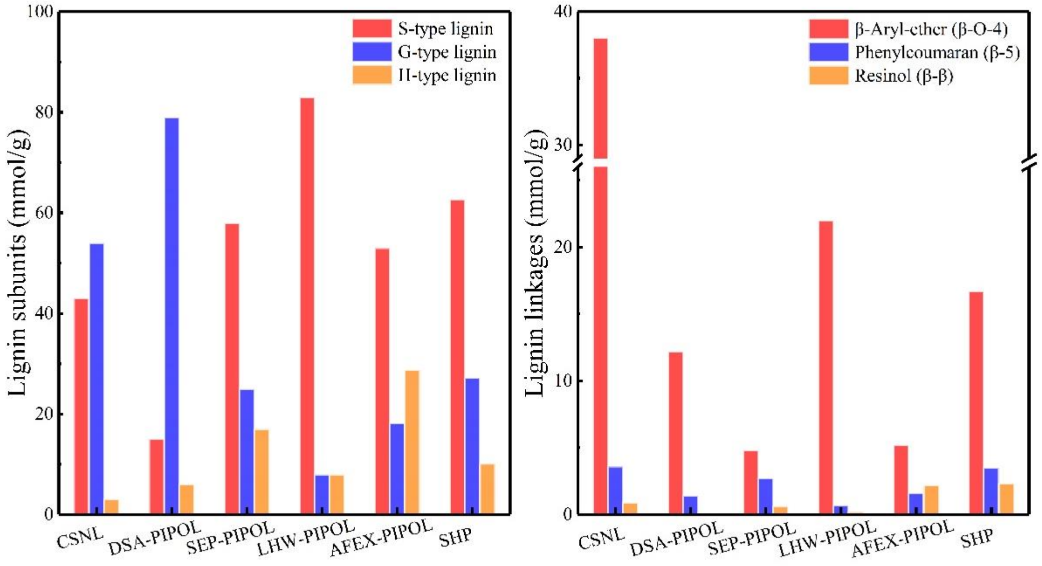

Supplementary Fig. 6 The subunits and linkages of the lignin samples. The soluble lignin was fractionated from the PIPOL-integrated biorefinery scenario with dilute sulfuric acid pretreatment (DSA), steam explosion pretreatment (SEP), liquid hot water pretreatment (LHW), ammonia fiber expansion (AFEX), sodium hydroxide pretreatment (SHP), respectively. PIPOL, 'plug-in processes of lignin'. CSNL, corn stover native lignin. S-, G-, and H- type represents syringyl, guaiacyl, and *p*-hydroxyphenylpropane unit of lignin, respectively.

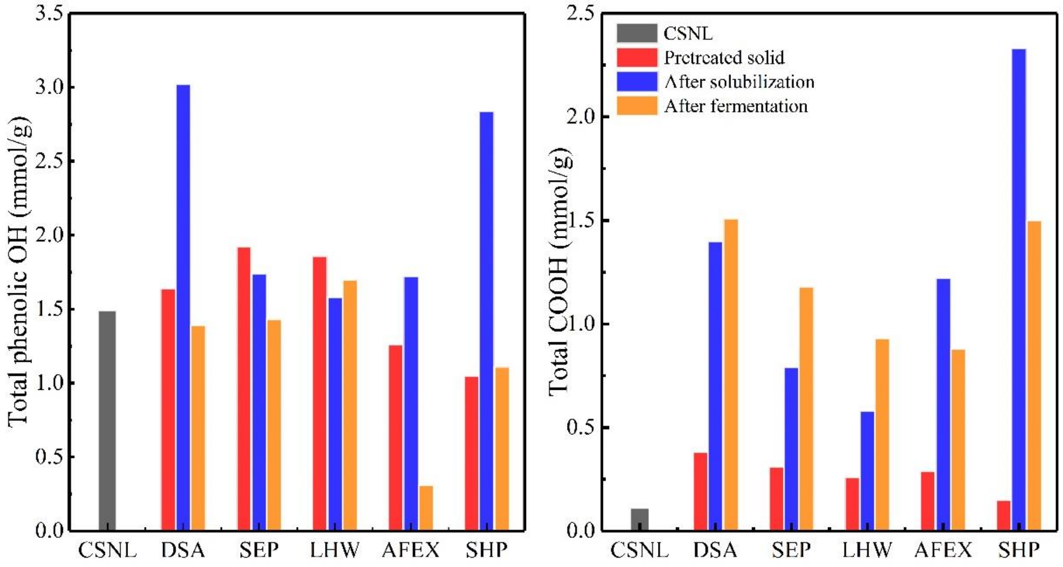

Supplementary Fig. 7 Total phenolic hydroxyl and carboxyl groups of the lignin samples. The lignin samples were prepared from the pretreated solid of leading pretreatment, and the soluble lignin streams after the solubilization and fermentation. CSNL, corn stover native lignin; DSA, dilute sulfuric acid pretreatment; SEP, steam explosion pretreatment; LHW, liquid hot water pretreatment; AFEX, ammonia fiber expansion; SHP, sodium hydroxide pretreatment.

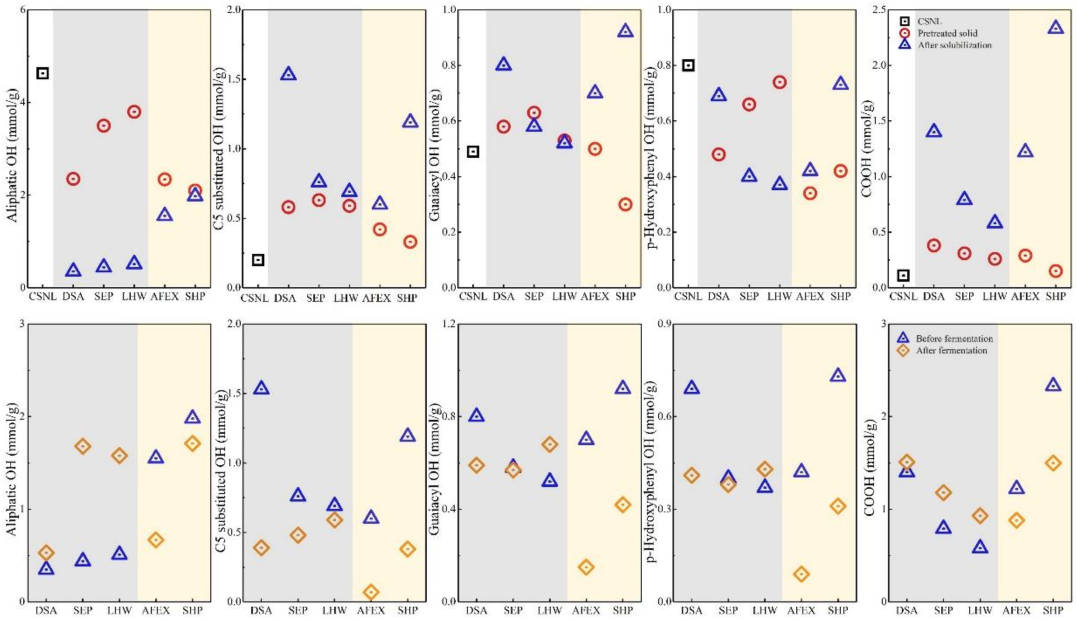

Supplementary Fig. 8 Functional hydrophilic groups (aliphatic hydroxyl, phenolic hydroxyl, and carboxyl groups) of the lignin samples. The lignin samples were prepared from the pretreated solid of leading pretreatment, and the soluble lignin streams after the solubilization and fermentation. CSNL, corn stover native lignin; DSA, dilute sulfuric acid pretreatment; SEP, steam explosion pretreatment; LHW, liquid hot water pretreatment; AFEX, ammonia fiber expansion; SHP, sodium hydroxide pretreatment. Gray mark represents the acidic pretreatment technologies while yellow mark represents the alkaline pretreatment technologies.

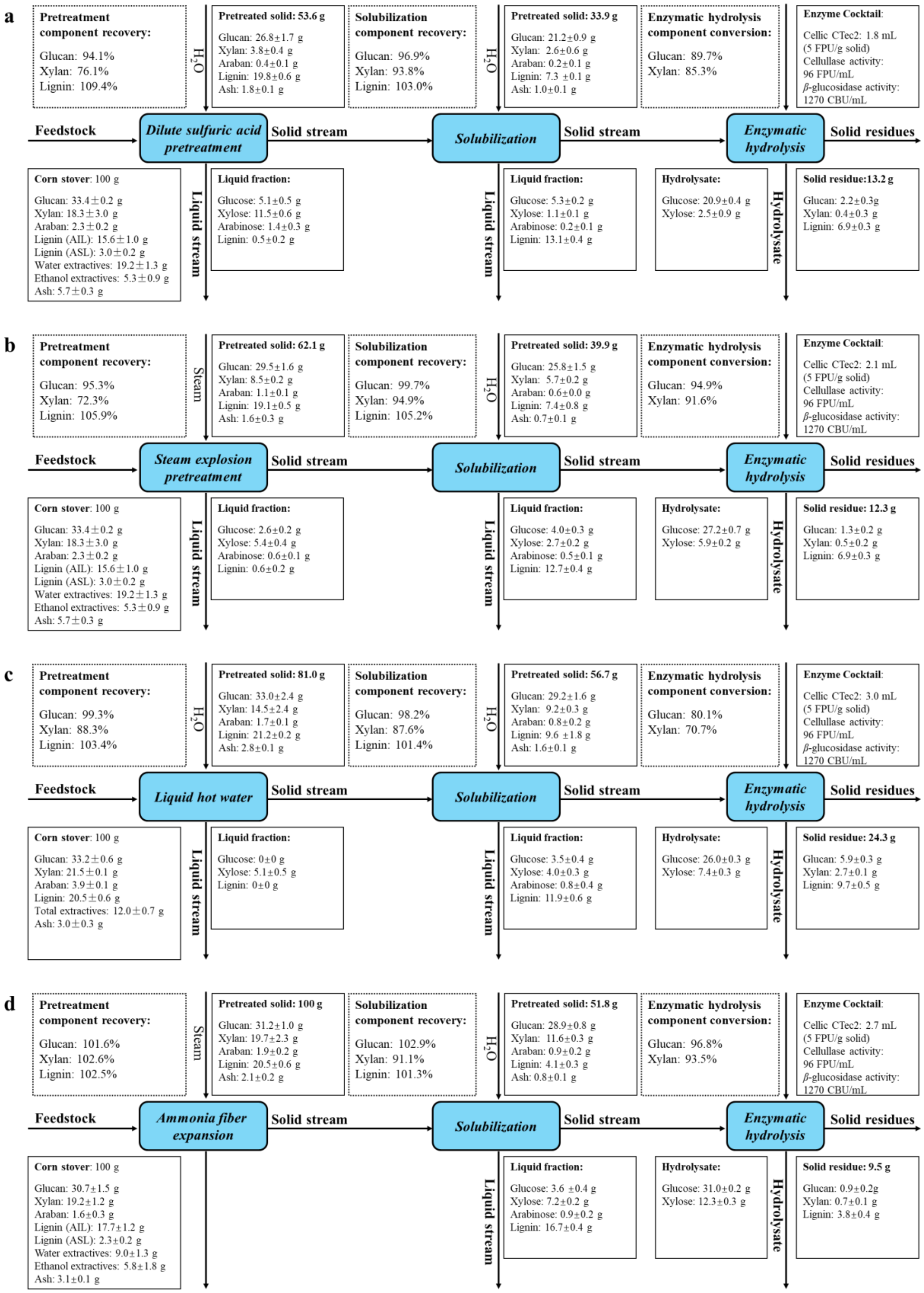
a
Pretreatment component recovery:
Glucan: 94.1%
Xylan: 76.1%
Lignin: 109.4%
H2O
Pretreated solid: 53.6 g
Glucan: 26.8±1.7 g
Xylan: 3.8±0.4 g
Araban: 0.4±0.1 g
Lignin: 19.8±0.6 g
Ash: 1.8±0.1 g
Solubilization component recovery:
Glucan: 96.9%
Xylan: 93.8%
Lignin: 103.0%
H2O
Pretreated solid: 33.9 g
Glucan: 21.2±0.9 g
Xylan: 2.6±0.6 g
Araban: 0.2±0.1 g
Lignin: 7.3 ±0.1 g
Ash: 1.0±0.1 g
Enzymatic hydrolysis component conversion:
Glucan: 89.7%
Xylan: 85.3%
Enzyme Cocktail:
Cellic CTec2: 1.8 mL
(5 FPU/g solid)
Cellulase activity:
96 FPU/mL
β-glucosidase activity:
1270 CBU/mL
Feedstock
Dilute sulfuric acid pretreatment
Solid stream
Solubilization
Solid stream
Enzymatic hydrolysis
Solid residues
Corn stover: 100 g
Glucan: 33.4±0.2 g
Xylan: 18.3±3.0 g
Araban: 2.3±0.2 g
Lignin (AIL): 15.6±1.0 g
Lignin (ASL): 3.0±0.2 g
Water extractives: 19.2±1.3 g
Ethanol extractives: 5.3±0.9 g
Ash: 5.7±0.3 g
Liquid stream
Liquid fraction:
Glucose: 5.1±0.5 g
Xylose: 11.5±0.6 g
Arabinose: 1.4±0.3 g
Lignin: 0.5±0.2 g
Liquid stream
Liquid fraction:
Glucose: 5.3±0.2 g
Xylose: 1.1±0.1 g
Arabinose: 0.2±0.1 g
Lignin: 13.1±0.4 g
Hydrolysate:
Glucose: 20.9±0.4 g
Xylose: 2.5±0.9 g
Hydrolysate
Solid residue:13.2 g
Glucan: 2.2±0.3g
Xylan: 0.4±0.3 g
Lignin: 6.9±0.3 g
b
Pretreatment component recovery:
Glucan: 95.3%
Xylan: 72.3%
Lignin: 105.9%
Steam
Pretreated solid: 62.1 g
Glucan: 29.5±1.6 g
Xylan: 8.5±0.2 g
Araban: 1.1±0.1 g
Lignin: 19.1±0.5 g
Ash: 1.6±0.3 g
Solubilization component recovery:
Glucan: 99.7%
Xylan: 94.9%
Lignin: 105.2%
H2O
Pretreated solid: 39.9 g
Glucan: 25.8±1.5 g
Xylan: 5.7±0.2 g
Araban: 0.6±0.0 g
Lignin: 7.4±0.8 g
Ash: 0.7±0.1 g
Enzymatic hydrolysis component conversion:
Glucan: 94.9%
Xylan: 91.6%
Enzyme Cocktail:
Cellic CTec2: 2.1 mL
(5 FPU/g solid)
Cellulase activity:
96 FPU/mL
β-glucosidase activity:
1270 CBU/mL
Feedstock
Steam explosion pretreatment
Solid stream
Solubilization
Solid stream
Enzymatic hydrolysis
Solid residues
Corn stover: 100 g
Glucan: 33.4±0.2 g
Xylan: 18.3±3.0 g
Araban: 2.3±0.2 g
Lignin (AIL): 15.6±1.0 g
Lignin (ASL): 3.0±0.2 g
Water extractives: 19.2±1.3 g
Ethanol extractives: 5.3±0.9 g
Ash: 5.7±0.3 g
Liquid stream
Liquid fraction:
Glucose: 2.6±0.2 g
Xylose: 5.4±0.4 g
Arabinose: 0.6±0.1 g
Lignin: 0.6±0.2 g
Liquid stream
Liquid fraction:
Glucose: 4.0±0.3 g
Xylose: 2.7±0.2 g
Arabinose: 0.5±0.1 g
Lignin: 12.7±0.4 g
Hydrolysate:
Glucose: 27.2±0.7 g
Xylose: 5.9±0.2 g
Hydrolysate
Solid residue: 12.3 g
Glucan: 1.3±0.2 g
Xylan: 0.5±0.2 g
Lignin: 6.9±0.3 g
c
Pretreatment component recovery:
Glucan: 99.3%
Xylan: 88.3%
Lignin: 103.4%
H2O
Pretreated solid: 81.0 g
Glucan: 33.0±2.4 g
Xylan: 14.5±2.4 g
Araban: 1.7±0.1 g
Lignin: 21.2±0.2 g
Ash: 2.8±0.1 g
Solubilization component recovery:
Glucan: 98.2%
Xylan: 87.6%
Lignin: 101.4%
H2O
Pretreated solid: 56.7 g
Glucan: 29.2±1.6 g
Xylan: 9.2±0.3 g
Araban: 0.8±0.2 g
Lignin: 9.6 ±1.8 g
Ash: 1.6±0.1 g
Enzymatic hydrolysis component conversion:
Glucan: 80.1%
Xylan: 70.7%
Enzyme Cocktail:
Cellic CTec2: 3.0 mL
(5 FPU/g solid)
Cellulase activity:
96 FPU/mL
β-glucosidase activity:
1270 CBU/mL
Feedstock
Liquid hot water
Solid stream
Solubilization
Solid stream
Enzymatic hydrolysis
Solid residues
Corn stover: 100 g
Glucan: 33.2±0.6 g
Xylan: 21.5±0.1 g
Araban: 3.9±0.1 g
Lignin: 20.5±0.6 g
Total extractives: 12.0±0.7 g
Ash: 3.0±0.3 g
Liquid stream
Liquid fraction:
Glucose: 0±0 g
Xylose: 5.1±0.5 g
Lignin: 0±0 g
Liquid stream
Liquid fraction:
Glucose: 3.5±0.4 g
Xylose: 4.0±0.3 g
Arabinose: 0.8±0.4 g
Lignin: 11.9±0.6 g
Hydrolysate:
Glucose: 26.0±0.3 g
Xylose: 7.4±0.3 g
Hydrolysate
Solid residue: 24.3 g
Glucan: 5.9±0.3 g
Xylan: 2.7±0.1 g
Lignin: 9.7±0.5 g
d
Pretreatment component recovery:
Glucan: 101.6%
Xylan: 102.6%
Lignin: 102.5%
Steam
Pretreated solid: 100 g
Glucan: 31.2±1.0 g
Xylan: 19.7±2.3 g
Araban: 1.9±0.2 g
Lignin: 20.5±0.6 g
Ash: 2.1±0.2 g
Solubilization component recovery:
Glucan: 102.9%
Xylan: 91.1%
Lignin: 101.3%
H2O
Pretreated solid: 51.8 g
Glucan: 28.9±0.8 g
Xylan: 11.6±0.3 g
Araban: 0.9±0.2 g
Lignin: 4.1±0.3 g
Ash: 0.8±0.1 g
Enzymatic hydrolysis component conversion:
Glucan: 96.8%
Xylan: 93.5%
Enzyme Cocktail:
Cellic CTec2: 2.7 mL
(5 FPU/g solid)
Cellulase activity:
96 FPU/mL
β-glucosidase activity:
1270 CBU/mL
Feedstock
Ammonia fiber expansion
Solid stream
Solubilization
Solid stream
Enzymatic hydrolysis
Solid residues
Corn stover: 100 g
Glucan: 30.7±1.5 g
Xylan: 19.2±1.2 g
Araban: 1.6±0.3 g
Lignin (AIL): 17.7±1.2 g
Lignin (ASL): 2.3±0.2 g
Water extractives: 9.0±1.3 g
Ethanol extractives: 5.8±1.8 g
Ash: 3.1±0.1 g
Liquid fraction:
Glucose: 3.6 ±0.4 g
Xylose: 7.2±0.2 g
Arabinose: 0.9±0.2 g
Lignin: 16.7±0.4 g
Liquid stream
Hydrolysate:
Glucose: 31.0±0.2 g
Xylose: 12.3±0.3 g
Hydrolysate
Solid residue: 9.5 g
Glucan: 0.9±0.2g
Xylan: 0.7±0.1 g
Lignin: 3.8±0.4 g

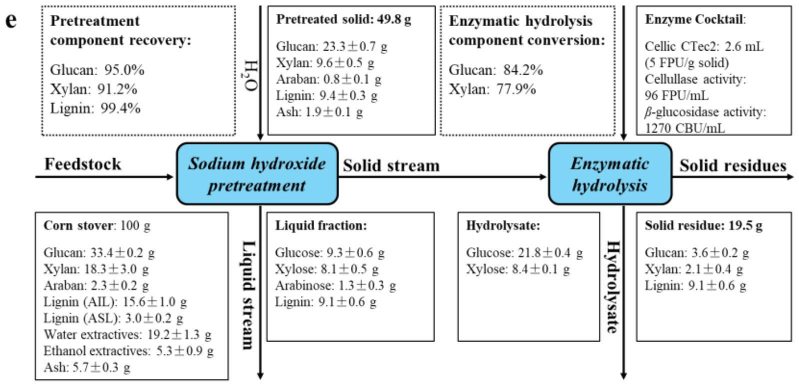

Supplementary Fig. 9 Mass balance for the whole processes of each biorefinery scenario. a, PIPOL-integrated biorefinery scenario with dilute sulfuric acid pretreatment (DSA); b, PIPOL-integrated biorefinery scenario with steam explosion pretreatment (SEP); c, PIPOL-integrated biorefinery scenario with liquid hot water pretreatment (LHW); d, PIPOL-integrated biorefinery scenario with ammonia fiber expansion (AFEX); e, PIPOL-integrated biorefinery scenario with sodium hydroxide pretreatment (SHP). PIPOL, ‘plug-in processes of lignin (PIPOL)’. Corn stover used for DSA, SEP and SHP were harvested from the suburb of Comanche, TX, United States. Corn stover used for AFEX and LHW was provided by Michigan State University and Montana State University, respectively.

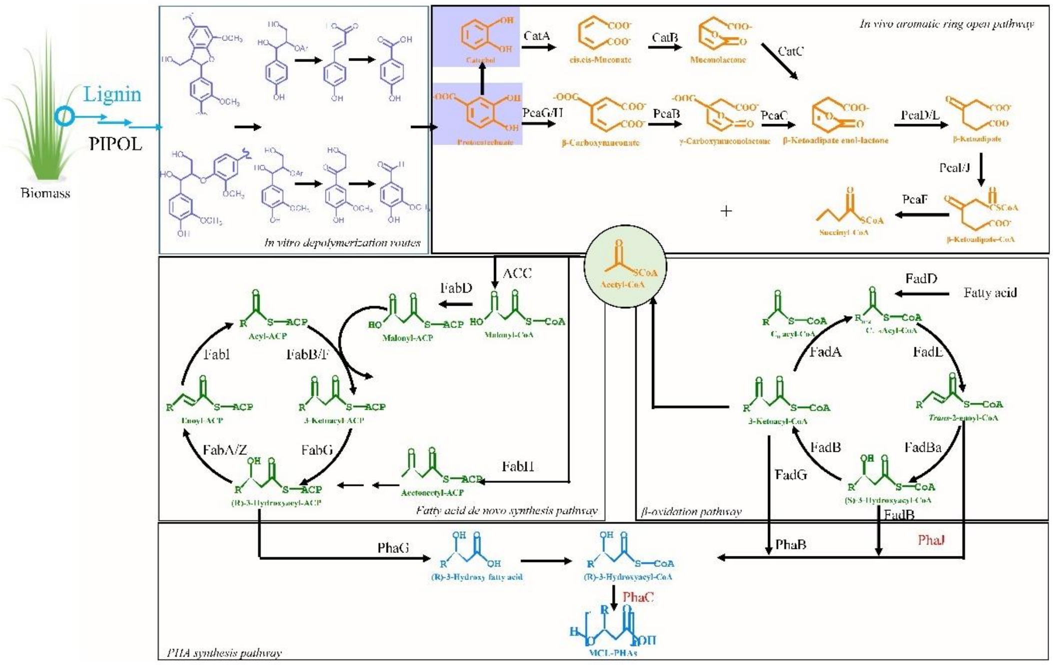

Supplementary Fig. 10 Metabolism pathways of lignin and aromatics for the biosynthesis of polyhydroxyalkanoates (PHAs). This pathway represents the ‘biological funnel’ of lignin valorization by engineered *Pseudomonas putida* KT2440. PIPOL represents ‘plug-in processes of lignin’.

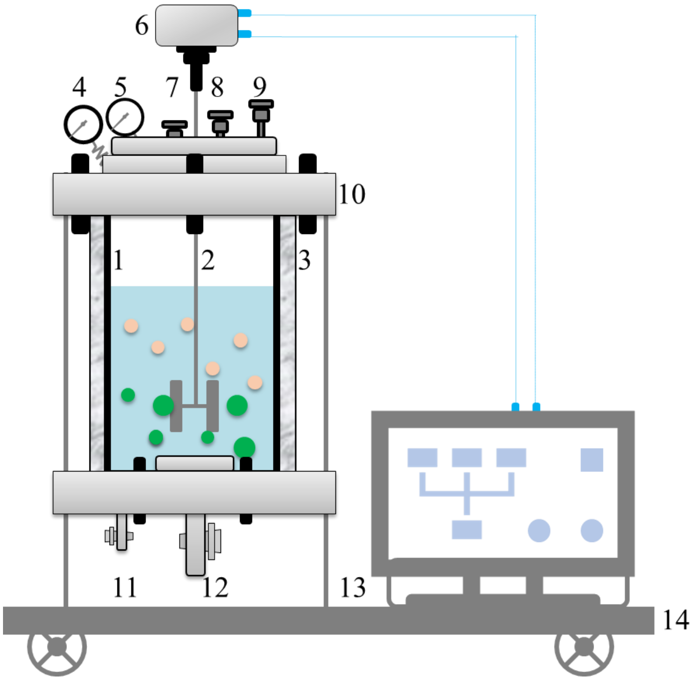

Supplementary Fig. 11 Schematic diagram of the pretreatment reactor systems. 1, Reactor chamber; 2, Anchor stirrer; 3, Heating jacket; 4, Temperature gauge; 5, Pressure gauge; 6, Drive system; 7, Liquid sample valve; 8, Gas release valve; 9, Inlet valve; 10, Rupture disc assembly; 11, Bottom drain valve; 12, Bottom outlet valve; 13, Reactor controller; 14, Cart stand

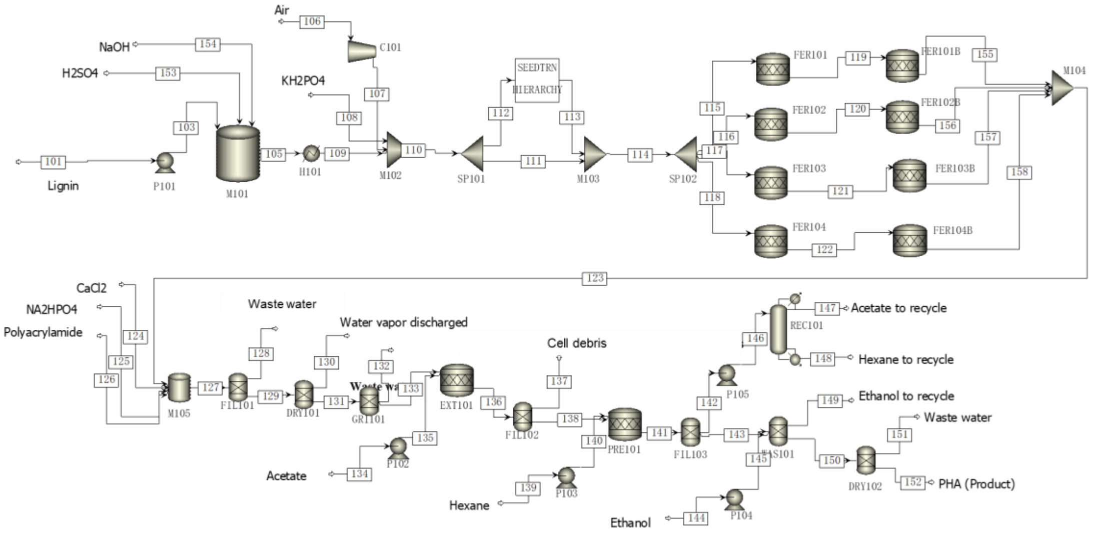

Supplementary Fig. 12 Aspen Plus process flow diagram of the ‘plug-in processes of lignin (PIPOL)’ of biological lignin valorization to polyhydroxyalkanoates (PHAs). The process includes lignin treatment, PHA fermentation, cell recovery, and PHA extraction.

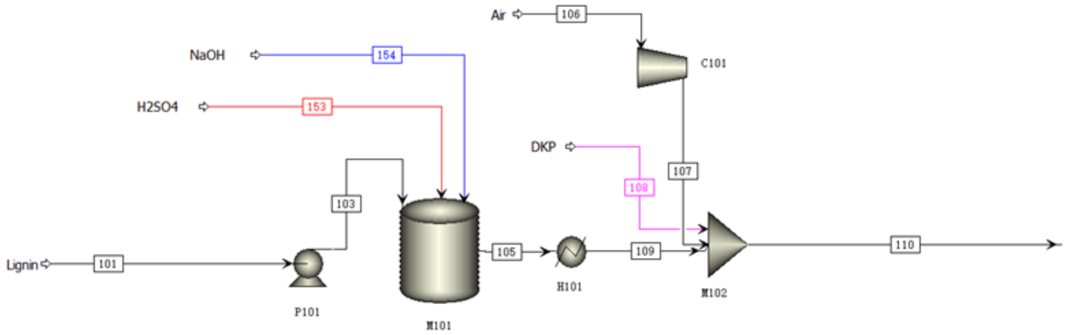

Supplementary Fig. 13 Aspen model of the flow diagram of the lignin stream conditioning.

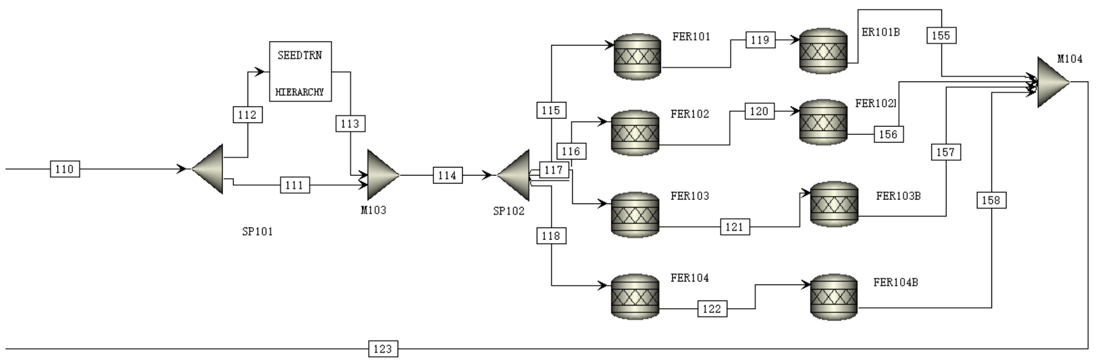

Supplementary Fig. 14 Aspen model of the flow diagram of the polyhydroxyalkanoate fermentation process using soluble lignin.

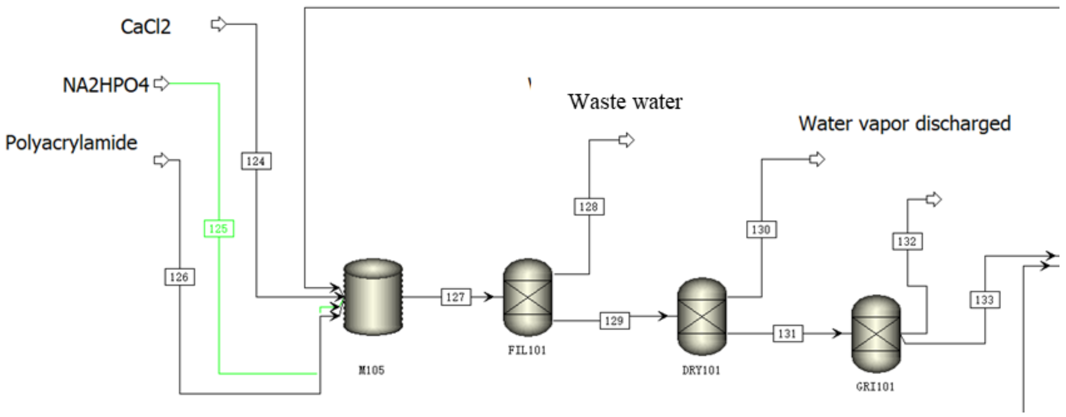

Supplementary Fig. 15 Aspen model of the flow diagram of the cell harvesting operation after polyhydroxyalkanoate fermentation.

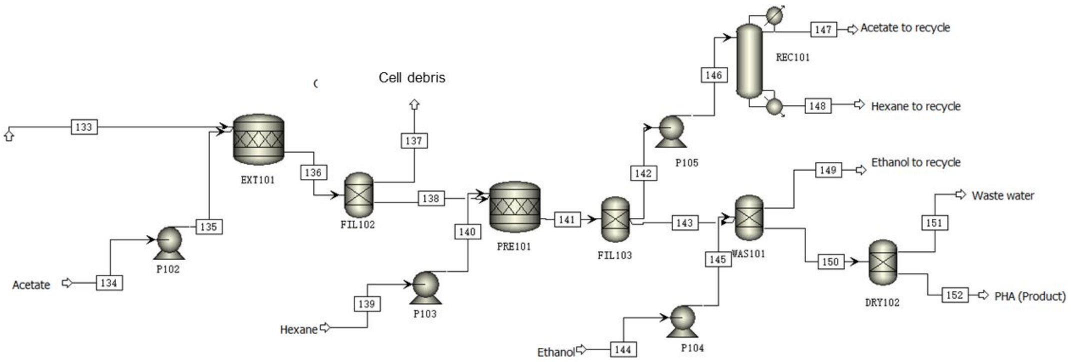

Supplementary Fig. 16 Aspen model of the flow diagram of the extraction operation of polyhydroxyalkanoates.

Supplementary Table 1 The conditions of leading pretreatment employed to deconstruct corn stover biomass

Experiment No.	Pretreatment	Temperature (°C)	Time (min)	Chemicals	Solid loading
Case 1	Dilute sulfuric acid	150	10	0.2% H_2SO_4	10%
Case 2	Steam explosion	200	10	None	40%
Case 3	Liquid hot water	160	5	None	10%
Case 4	Ammonia fiber expansion	140	15	1:1 ammonia loading	40%
Case 5	Alkaline pretreatment	150	10	1.0% NaOH	10%

Chemicals used in pretreatment were based on the total weight of solid and liquid; Solid loading was calculated based on the mass fraction of the mixture, % (w/w)

Supplementary Table 2 Compositions of corn stover feedstock used in biorefinery

	Corn stover 1		Corn stover 2	
Composition	Content (%)	SD	Content (%)	SD
Glucan	33.4	0.2	30.7	1.5
Xylan	18.3	3.0	19.2	1.2
Arabinan	2.3	0.2	1.6	0.3
Acid insoluble lignin (AIL)	15.6	1.0	17.7	1.2
Acid soluble lignin (ASL)	3.0	0.2	2.3	0.2
Water extractives	19.2	1.3	9.0	1.3
Ethanol extractives	5.3	0.9	5.8	1.8
Ash	5.7	0.3	3.1	0.1

Corn stover 1 was harvested from the suburb of Comanche, TX, United States; Corn stover 2 was provided by Michigan State University, MI, United States; SD represents the standard deviation

Supplementary Table 3 Financial assumptions and design basis [7-12]

Case	Value
Plant life	30 years
Cost year (dollar unit)	2014 dollars
Capacity factor	90%
Discount rate	10%
General plant depreciation	200% declining balance
General plant recovery period	7 years
Steam plant depreciation	150% declining balance
Steam plant recovery period	20 years
Federal tax rate	35%
Financing	40% equity
Loan terms	10 year loan at 8% annual percentage rate
Construction period	3 years
First 12 months' expenditures	8%
Next 12 months' expenditures	60%
Last 12 months' expenditures	32%
Working capital	5% of fixed capital investment
Start-up time	3 months
Revenues during start-up	50%
Variable costs during start-up	75%
Fixed costs during start-up	100%
Lignin ($/ton)	55.8
H_2SO_4 ($/ton)	81.39
NaOH ($/ton)	135.65
Ethyl acetate ($/ton)	1100
Hexane ($/lb)	0.5394
Ethanol ($/gal)	2.051

Supplementary Table 4 Mass flow of key streams in the conditioning and fermentation

Component	Units	101	109	110	114	123
Total flow	kg/hr	22375.437	71664.840	83736.240	83737.440	83737.440
Water	kg/hr	7836.510	57122.310	57122.310	57122.740	57126.480
Ethanol	kg/hr	3.493	3.493	3.493	3.493	3.493
Glucose (SS)	kg/hr	17.822	17.822	17.822	16.040	4.812
Galactose (SS)	kg/hr	26.082	26.082	26.082	26.082	26.082
Mannose (SS)	kg/hr	10.944	10.944	10.944	10.944	10.944
Xylose (SS)	kg/hr	26.527	26.527	26.527	26.527	26.527
Arabinose (SS)	kg/hr	5.016	5.016	5.016	5.016	5.016
Glucooligomers (SS)	kg/hr	31.788	31.788	31.788	31.788	31.788
Galactooligomers (SS)	kg/hr	0.652	0.652	0.652	0.652	0.652
Mannooligomers (SS)	kg/hr	0.274	0.274	0.274	0.274	0.274
Extractives (SS)	kg/hr	278.064	278.064	278.064	278.064	278.064
Lignisol (SS)	kg/hr	14.965	14.965	14.965	14.965	14.965
HMF	kg/hr	8.404	8.404	8.404	8.404	8.404
Furfurals	kg/hr	3.331	3.331	3.331	3.331	3.331
Lactic acid	kg/hr	38.607	38.607	38.607	38.607	38.607
Xylitol	kg/hr	18.553	18.553	18.553	18.553	18.553
Glycerol	kg/hr	0.528	0.528	0.528	0.528	0.528
Succinic acid	kg/hr	1.848	1.848	1.848	1.848	1.848
Ammonia	kg/hr	-	3.600	3.600	3.600	3.600
Ammonium sulfate	kg/hr	56.560	56.560	56.560	56.560	56.560
Ammonium acetate	kg/hr	42.422	42.422	42.422	42.422	42.422
DAP	kg/hr	2.970	2.970	2.970	74.346	74.346
O_2	kg/hr	0.328	0.328	2795.332	2795.332	168.410
N_2	kg/hr	0.585	0.585	9205.581	9205.581	9205.581
CO_2	kg/hr	-	-	-	-	4464.517
PHA (IS)	kg/hr	-	-	-	-	2263.076
P. Putida	kg/hr	-	-	-	2.662	2.662
Cell mass (IS)	kg/hr	-	-	-	-	1775.836
Cellulose (IS)	kg/hr	768.559	768.559	768.559	768.559	768.559
Galactan (IS)	kg/hr	18.218	18.218	18.218	18.218	18.218
Mannan (IS)	kg/hr	7.644	7.644	7.644	7.644	7.644
Xylan (IS)	kg/hr	478.485	478.485	478.485	478.485	478.485
Arabinan (IS)	kg/hr	58.310	58.310	58.310	58.310	58.310
Lignin (IS)	kg/hr	7336.278	7336.278	7336.278	7336.278	1467.256
Protein (IS)	kg/hr	1684.852	1684.852	1684.852	1684.769	1684.769
Ash	kg/hr	2415.700	2415.700	2415.700	2415.700	2415.700
Enzyme (IS)	kg/hr	306.269	306.269	306.269	306.269	306.269
Other IS	kg/hr	874.545	874.545	874.545	877.207	877.207

Supplementary Table 5 Mass flow of key streams in the cell harvesting section

Component	Units	123	127	129	131	133
Total flow	kg/hr	83737.44	85198.71	20690.302	12129.899	12121.330
Water	kg/hr	57126.48	57126.480	8568.972	8.569	8.569
Ethanol	kg/hr	3.49	3.493	-	-	-
Glucose (SS)	kg/hr	4.81	4.812	-	-	-
Galactose (SS)	kg/hr	26.08	26.082	-	-	-
Mannose (SS)	kg/hr	10.94	10.944	-	-	-
Xylose (SS)	kg/hr	26.53	26.527	-	-	-
Arabinose (SS)	kg/hr	5.02	5.016	-	-	-
Glucooligomers (SS)	kg/hr	31.788	31.788	-	-	-
Galactooligomers (SS)	kg/hr	0.652	0.652	-	-	-
Mannooligomers (SS)	kg/hr	0.274	0.274	-	-	-
Extractives (SS)	kg/hr	278.064	278.064	-	-	-
Lignisol (SS)	kg/hr	14.965	14.965	-	-	-
HMF	kg/hr	8.404	8.404	-	-	-
Furfurals	kg/hr	3.331	3.331	-	-	-
Lactic acid	kg/hr	38.607	38.607	-	-	-
Xylitol	kg/hr	18.553	18.553	-	-	-
Glycerol	kg/hr	0.528	0.528	-	-	-
Succinic acid	kg/hr	1.848	1.848	-	-	-
Ammonia	kg/hr	3.600	3.600	-	-	-
Ammonium sulfate	kg/hr	56.560	56.560	-	-	-
Ammonium acetate	kg/hr	42.422	42.422	-	-	-
DAP	kg/hr	74.346	74.346	-	-	-
O2	kg/hr	168.410	168.410	-	-	-
N2	kg/hr	9205.581	9205.581	-	-	-
CO2	kg/hr	4464.517	4464.517	-	-	-
PHA (IS)	kg/hr	2263.076	2263.076	2263.076	2263.076	2263.076
Cell mass (IS)	kg/hr	1775.836	1775.836	1775.836	1775.836	1775.836
Cellulose (IS)	kg/hr	768.559	768.559	768.559	768.559	768.559
Galactan (IS)	kg/hr	18.218	18.218	18.218	18.218	18.218
Mannan (IS)	kg/hr	7.644	7.644	7.644	7.644	7.644
Xylan (IS)	kg/hr	478.485	478.485	478.485	478.485	478.485
Arabinan (IS)	kg/hr	58.310	58.310	58.310	58.310	58.310
Lignin (IS)	kg/hr	1467.256	1467.256	1467.256	1467.256	1467.256
Protein (IS)	kg/hr	1684.769	1684.769	1684.769	1684.769	1684.769
Ash	kg/hr	2415.700	2415.700	2415.700	2415.700	2415.700
Enzyme (IS)	kg/hr	306.269	306.269	306.269	306.269	306.269
Other IS	kg/hr	877.207	877.207	877.207	877.207	877.207

Supplementary Table 6 Mass flow of key streams in the polyhydroxyalkanoate extraction section

Component	Units	136	138	141	143	150	152
Total flow	kg/hr	13475.210	3616.958	6576.328	2271.645	2271.645	2263.076
Water	kg/hr	8.569	8.569	8.569	8.569	8.569	
Ethanol	kg/hr	-	-	-	-	-	-
Ethyl acetate	gal/hr	1345.313	1345.313	1345.313	-	-	-
Hexane	gal/hr	-	-	2959.370	-	-	-
PHA (IS)	kg/hr	2263.076	2263.076	2263.076	2263.076	2263.076	2263.076
Cell mass (IS)	kg/hr	1775.836	-	-	-	-	-
Cellulose (IS)	kg/hr	768.559	-	-	-	-	-
Galactan (IS)	kg/hr	18.218	-	-	-	-	-
Mannan (IS)	kg/hr	7.644	-	-	-	-	-
Xylan (IS)	kg/hr	478.485	-	-	-	-	-
Arabinan (IS)	kg/hr	58.310	-	-	-	-	-
Lignin (IS)	kg/hr	1467.256	-	-	-	-	-
Protein (IS)	kg/hr	1684.769	-	-	-	-	-
Ash	kg/hr	2415.700	-	-	-	-	-
Enzyme (IS)	kg/hr	306.269	-	-	-	-	-
Other IS	kg/hr	877.207	-	-	-	-	-

Supplementary Table 7 Mass flow of H_2SO_4 and NaOH consumed in PIPOL-integrated biorefinery

Component	Units	Scenario 1	Scenario 2	Scenario 3	Scenario 4	Scenario 5
H_2SO4	kg/hr	451.32	868.41	778.15	1711.92	1637.22
NaOH	kg/hr	367.28	834.17	778.15	778.15	1671.46

Scenario 1-5 represents PIPOL-integrated biorefinery with dilute sulfuric acid pretreatment (DSA), steam explosion pretreatment (SEP), liquid hot water pretreatment (LHW), ammonia fiber expansion (AFEX), sodium hydroxide pretreatment (SHP), respectively. PIPOL, 'plug-in processes of lignin'.

Supplementary References

1. Lin, L. et al. Systems biology-guided biodesign of consolidated lignin conversion. *Green Chem* **18**, 5536-5547 (2016).
2. Meng, X.Z. et al. Determination of hydroxyl groups in biorefinery resources via quantitative (PNMR)-P-31-N- spectroscopy. *Nat Protoc* **14**, 2627-2647 (2019).
3. Pu, Y.Q., Cao, S.L. & Ragauskas, A.J. Application of quantitative P-31 NMR in biomass lignin and biofuel precursors characterization. *Energ Environ Sci* **4**, 3154-3166 (2011).
4. Yoo, C.G., Pu, Y.Q., Li, M. & Ragauskas, A.J. Elucidating Structural Characteristics of Biomass using Solution-State 2D NMR with a Mixture of Deuterated Dimethylsulfoxide and Hexamethylphosphoramide. *Chemsuschem* **9**, 1090-1095 (2016).
5. Liu, Z.H. et al. Defining lignin nanoparticle properties through tailored lignin reactivity by sequential organosolv fragmentation approach (SOFA). *Green Chem* **21**, 245-260 (2019).
6. Samuel, R., Pu, Y.Q., Raman, B. & Ragauskas, A.J. Structural Characterization and Comparison of Switchgrass Ball-milled Lignin Before and After Dilute Acid Pretreatment. *Appl Biochem Biotech* **162**, 62-74 (2010).
7. Laskar DD, Tucker MP, Chen X, Helms GL, Yang B. Noble-metal catalyzed hydrodeoxygenation of biomass-derived lignin to aromatic hydrocarbons. Green Chemistry **16**, 897-910 (2014)
8. Phillips S. Technoeconomic analysis of a lignocellulosic biomass indirect gasification process to make ethanol via mixed alcohols synthesis. Industrial & Engineering Chemistry Research **46**, 8887-8897 (2007).
9. Tao L, et al. Techno-economic analysis and life-cycle assessment of cellulosic isobutanol and comparison with cellulosic ethanol and n-butanol. Biofuels, Bioprod Biorefin **8**, 30-48 (2014).
10. Jones SB, Zhu Y. Preliminary economics for the production of pyrolysis oil from lignin in a cellulosic ethanol biorefinery. Report PNNL-18401 Pacific Northwest National Laboratory for US Department of Energy, (2009).
11. Shen RC, Tao L, Yang B. Techno-economic analysis of jet-fuel production from biorefinery waste lignin. Biofuel Bioprod Bior **13**, 486-501 (2019).
12. Humbird D, et al. Process design and economics for biochemical conversion of lignocellulosic biomass to ethanol: dilute-acid pretreatment and enzymatic hydrolysis of corn stover. National Renewable Energy Lab. (NREL), Golden, CO (United States) (2011).
